# Correcting the impact of docking pose generation error on binding affinity prediction

**DOI:** 10.1186/s12859-016-1169-4

**Published:** 2016-09-22

**Authors:** Hongjian Li, Kwong-Sak Leung, Man-Hon Wong, Pedro J. Ballester

**Affiliations:** 1 Department of Computer Science and Engineering, Chinese University of Hong Kong, Hong Kong, China; 2 Cancer Research Center of Marseille, INSERM U1068, Marseille, F-13009 France; 3 Institut Paoli-Calmettes, Marseille, F-13009 France; 4 Aix-Marseille Université, Marseille, F-13284 France; 5 CNRS UMR7258, Marseille, F-13009 France

**Keywords:** Molecular docking, Binding affinity, Drug discovery, Machine learning

## Abstract

**Background:**

Pose generation error is usually quantified as the difference between the geometry of the pose generated by the docking software and that of the same molecule co-crystallised with the considered protein. Surprisingly, the impact of this error on binding affinity prediction is yet to be systematically analysed across diverse protein-ligand complexes.

**Results:**

Against commonly-held views, we have found that pose generation error has generally a small impact on the accuracy of binding affinity prediction. This is also true for large pose generation errors and it is not only observed with machine-learning scoring functions, but also with classical scoring functions such as AutoDock Vina. Furthermore, we propose a procedure to correct a substantial part of this error which consists of calibrating the scoring functions with re-docked, rather than co-crystallised, poses. In this way, the relationship between Vina-generated protein-ligand poses and their binding affinities is directly learned. As a result, test set performance after this error-correcting procedure is much closer to that of predicting the binding affinity in the absence of pose generation error (i.e. on crystal structures). We evaluated several strategies, obtaining better results for those using a single docked pose per ligand than those using multiple docked poses per ligand.

**Conclusions:**

Binding affinity prediction is often carried out on the docked pose of a known binder rather than its co-crystallised pose. Our results suggest than pose generation error is in general far less damaging for binding affinity prediction than it is currently believed. Another contribution of our study is the proposal of a procedure that largely corrects for this error. The resulting machine-learning scoring function is freely available at http://istar.cse.cuhk.edu.hk/rf-score-4.tgz and http://ballester.marseille.inserm.fr/rf-score-4.tgz.

**Electronic supplementary material:**

The online version of this article (doi:10.1186/s12859-016-1169-4) contains supplementary material, which is available to authorized users.

## Background

Molecular docking tools are routinely utilised to predict the binding pose as well as the binding affinity of a ligand, usually a small organic molecule, bound to a target protein of interest. On one hand, the predicted pose suggests putative intermolecular interactions that can be helpful to understand the mechanism of protein-ligand binding. On the other hand, the predicted affinity prioritizes strong-binding ligands over weak-binding ones from a large library of compounds to evaluate.

A typical docking program implements a sampling algorithm to generate possible binding poses and a scoring function to estimate their binding affinity. The former operation is known as pose generation, and the latter is known as scoring. For example, modern docking tools such as AutoDock Vina [1] and idock [2] are currently capable of generating near-native poses with a redocking success rate of over 50 % on three diverse benchmarks [2].

Recent years have seen the emergence and prosperity of a new class of scoring functions that use machine learning techniques to increase the accuracy of binding affinity prediction (a first review on machine-learning scoring functions has now been published [3]). RF-Score [4] was the first machine-learning scoring function introducing a substantial improvement over classical scoring functions. Since then, several enhancements have been introduced, thereby resulting in RF-Score-v2 [5] and RF-Score-v3 [6], and other relevant studies [7]. RF-Score has been utilised [8] to successfully discover a large number of innovative binders of antibacterial DHQase2 targets, demonstrating its practical utility. To promote its use, RF-Score-v3 has been incorporated into a user-friendly webserver called istar [2], available at http://istar.cse.cuhk.edu.hk/idock, for large-scale docking-based prospective virtual screening. Furthermore, recent study [9] has investigated the benefit of training machine-learning scoring functions with low-quality structural and interaction data.

In prospective structure-based virtual screening [2], scoring of the docked poses of a molecule is required because the experimentally determined pose is not available in most cases. Therefore, accurate prediction of binding affinity of docked poses, rather than co-crystallised poses, is required for ranking compounds from screening libraries.

**Fig. 1:**
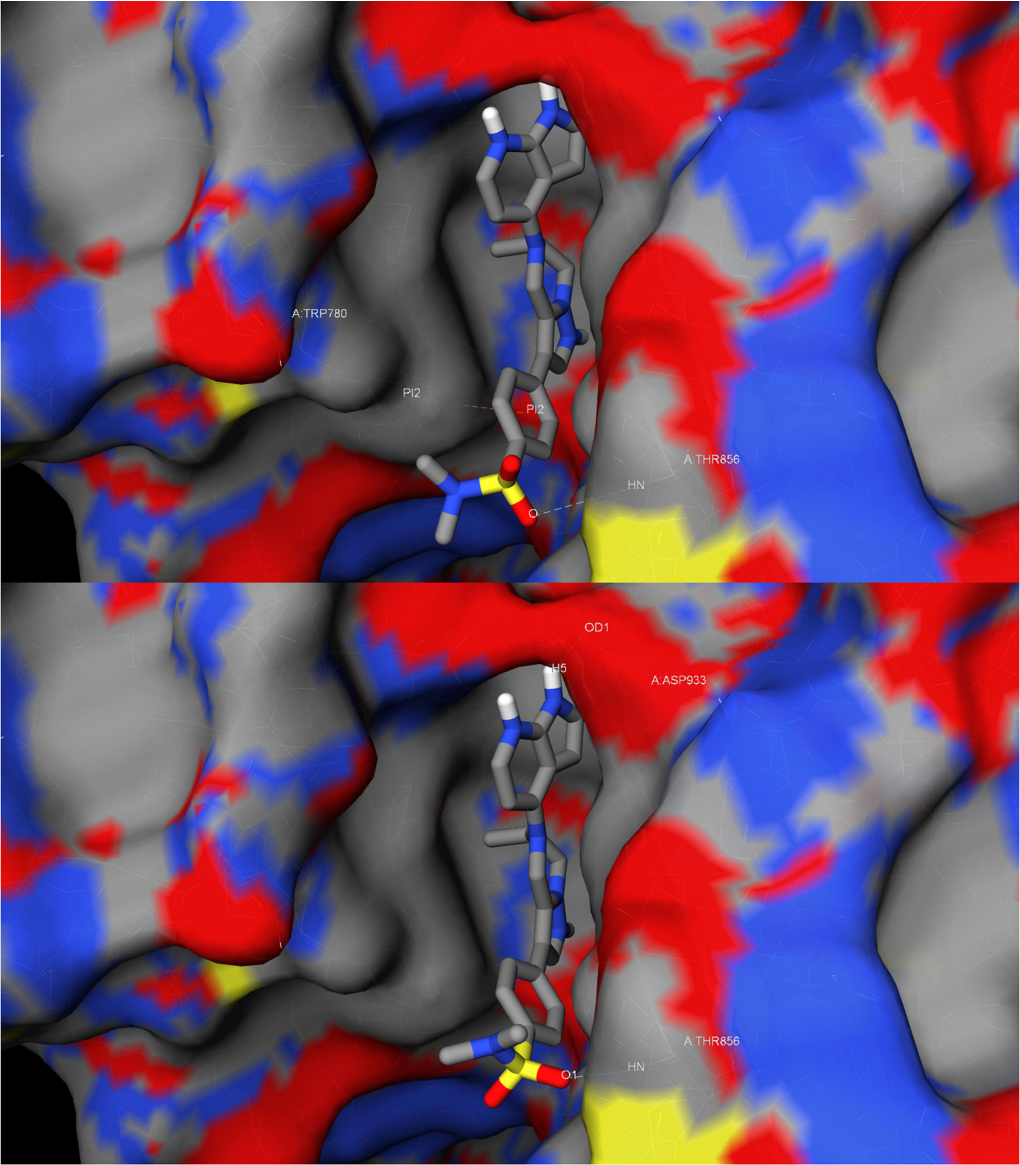
Example of pose generation error. *Top*: crystal structure of PI3K *α* in complex of a tetrahydropyrazolo[1,5-a]pyrazine codenamed 3K6 (PDB ID: 4WAF). *Bottom*: re-docked pose of 3K6, generated by idock [2]. Hydrogen bonds are rendered as dashed cyan lines, and *π* stackings are rendered as dashed pink lines. The RMSD (Root-Mean Square Deviation) between the co-crystallised pose and the re-docked pose of 3K6 is 1.15 Å, which is a quantitative measure of pose generation error. These two plots were created by iview [17], an interactive WebGL visualizer that circumvents the requirement of Java, yet supports the construction of macromolecular surface and the display of virtual reality effects and molecular interactions. iview is freely available at http://istar.cse.cuhk.edu.hk/iview/

Pose generation error is typically measured by comparing the geometry of the pose generated by the docking software and that of the same molecule co-crystallised with the considered protein (Fig. 1). The impact of this error on binding affinity prediction is yet to be systematically analysed across diverse protein-ligand complexes. In this study we investigate the impact of pose generation error on the predictive performance of both classical and machine-learning scoring functions, and propose a novel approach to correct such error. Furthermore, we release free software implementing these improvements.

## Methods

This section introduces and motivates the use of four scoring functions building upon AutoDock Vina, two benchmarks to evaluate and compare performance of these scoring functions, the performance metrics, and the experimental setup.

### Model 1 - AutoDock Vina

AutoDock Vina [1] was chosen as a baseline scoring function because of its popularity among the research community. Vina’s popularity roots in its substantial improvements on both the average accuracy of the binding pose prediction and the running speed. Its remarkable performance in pose generation as well as its open source nature are other appealing aspects of this widely-used tool.

Like all classical scoring functions [6], Vina assumes a predetermined functional form. Vina’s score for the *k*th pose of a molecule is given by the predicted free energy of binding to the target protein and computed as:

1 $$  e'_{k}=\frac{e_{k,inter}+e_{k,intra}-e_{1,intra}}{1+w_{6}N_{rot}}  $$

where

2 $$\begin{array}{@{}rcl@{}}  e_{k,inter} &=& w_{1} \cdot Gauss1_{k}  \\ &&+ w_{2} \cdot Gauss2_{k}  \\ &&+ w_{3} \cdot Repulsion_{k}  \\ &&+ w_{4} \cdot Hydrophobic_{k}  \\ &&+ w_{5} \cdot HBonding_{k} \end{array} $$

3 $$\begin{array}{@{}rcl@{}}  w_{1} &=& -0.035579  \\ w_{2} &=& -0.005156  \\ w_{3} &=& 0.840245  \\ w_{4} &=& -0.035069  \\ w_{5} &=& -0.587439  \\ w_{6} &=& 0.05846 \end{array} $$

$e^{\prime }_{k}$ is the predicted free energy of binding reported by the Vina software when scoring the *k*th docked pose. *e*_*k,inter*_ and *e*_*k,intra*_ are the inter-molecular and intra-molecular contributions, respectively, which have both the same functional form described in Eq. 2 but are summed over different atom pairs. The values for the six weights were calculated by OLS (Ordinary Least Squares) using a nonlinear optimisation algorithm as it has been the case in related force-field scoring functions [10], although this process was not fully disclosed in the original publication [1]. *N*_*rot*_ is the calculated number of rotatable bonds. The predicted free energy of binding in kcal/mol units was converted into *pK*_*d*_ units with *pK*_*d*_=−0.73349480509*e* so as to compare to binding affinities in *pK*_*d*_ or *pK*_*i*_ units. Mathematical expressions and further explanations can be found in [2].

Unlike our previous study [6] on scoring crystal poses, where *k*=1 because only the crystal pose was considered, this study aims at training and testing on docked poses, so *k* will range from 1 to 9 depending on the specific pose to use for each molecule (Vina returns a maximum of 9 poses per docking run). Thus *e*_*k,intra*_ and *e*_1,*intra*_ do not necessarily cancel out. As a result, the five terms from *e*_*k,intra*_ were considered as additional features in models 2, 3 and 4.

### Model 2 - MLR::Vina

This model retains the 11 unweighted Vina terms (5 from *e*_*k,inter*_, 5 from *e*_*k,intra*_, and *N*_*rot*_) as features, but changes the regression method to multiple linear regression (MLR), a regression model commonly adopted by classical scoring functions, such as empirical scoring functions. The use of MLR implies an additive functional form and therefore MLR::Vina is a classical scoring function [6].

Vina’s scoring function is not exactly a sum of energetic terms because *w*_6_≠0 (although the denominator of Eq. 1 is close to 1 because of the low value of *w*_6_. In order to make the problem amenable to MLR, we performed a grid search on *w*_6_ and thereafter ran MLR on the remaining weights. More precisely, we sampled 101 values for *w*_6_ from 0 to 1 with a step size of 0.01. Interestingly we found that the *w*_6_ values of the best models were always between 0.000 and 0.030. Then we again sampled 31 values for *w*_6_ in this range with a step size of 0.001, and used the *w*_6_ value that resulted in the lowest RMSE (Root Mean Square Error) on the test set.

### Model 3 - RF::Vina

This model also retains the 11 unweighted Vina terms as features, but changes the regression method to Random Forest (RF) [11], so as to implicitly learn the functional form from the data. Hence this model circumvents the modelling assumption of a predetermined functional form and thus allows to investigate the impact of such modelling assumption by comparing RF::Vina to MLR::Vina. Besides RF, other machine learning techniques such as SVR (Support Vector Regression) [12] can certainly be applied to this problem, although this is out of the scope of this study.

A RF is an ensemble of different decision trees randomly generated from the same training data via bootstraping [11]. RF trains its constituent trees using the CART algorithm [13], and selects the best data split at each node of the tree from a typically small number (mtry) of randomly chosen features. In regression applications, the RF prediction is given by arithmetic mean of all the individual tree predictions in the forest.

For each value of the mtry parameter from 1 to all 11 features, we built a RF model with 500 trees, as we and others [14] have not observed any substantial gain in performance by training RF with a higher number of trees on this class of problems. The selected model was the one that led to the lowest RMSE on a subset of training data of each tree collectively known as the OOB (Out of Bag) data. Because RF is stochastic, this process was repeated ten times with ten different random seeds. The predictive performance was reported for the RF with the best seed that resulted in the lowest RMSE on the test set. Further details on RF model building in this context can be found in [6].

### Model 4 - RF::VinaElem

This model retains RF as the regression method, but expands the feature set to 47 features by adding the 36 RF-Score [4] features. Like in the training process of RF::Vina, the same ten seeds were used, and for a given random seed, a RF model for each mtry value from 1 to 47 was built and that with the lowest RMSE on OOB data was selected. The predictive performance was reported for the RF with the best seed that led to the lowest RMSE on the test set.

RF-Score features are defined as the occurrence count of intermolecular contacts between elemental atom types *i* and *j*, as shown in Eqs. 4 and 5, where *d*_*kl*_ is the Euclidean distance between the *k*th protein atom of type *j* and the *l*th ligand atom of type *i* calculated from a structure; *K*_*j*_ is the total number of protein atoms of type *j* (*#*{*j*}=4, considered protein atom types are C, N, O, S) and *L*_*i*_ is the total number of ligand atoms of type *i* (*#*{*i*}=9, considered ligand atom types are C, N, O, F, P, S, Cl, Br, I); $\mathcal {H}$ is the Heaviside step function that counts contacts within a neighbourhood of *d*_*cutoff*_ Å. For instance, *x*_7,8_ is the number of occurrences of ligand nitrogen atoms (*i*=7) hypothetically interacting with protein oxygen atoms (*j*=8) within a chosen neighbourhood. Full details on RF-Score features are available in [4, 12].

4 $$  x_{ij}=\sum\limits_{k=1}^{K_{j}}\sum\limits_{l=1}^{L_{i}}\mathcal{H}(d_{cutoff}-d_{kl})  $$

5 $$  \mathbf x=\{x_{ij}\}\in N^{36}  $$

### PDBbind v2007 benchmark

We adopted the PDBbind v2007 benchmark [15], arguably the most widely used [6, 7] for binding affinity prediction of diverse complexes. Its test set comprises 195 diverse complexes from the core set, whereas its training set comprises 1105 non-overlapping complexes from the refined set. Both the test and training sets come with measured binding affinities spanning more than 12 orders of magnitude. This benchmark has the advantage of permitting a direct comparison against the same four models that were trained and tested on crystal poses [6] of this benchmark.

### PDBbind v2013 blind benchmark

We also adopted the PDBbind v2013 blind benchmark [6], a recently proposed new benchmark mimicking a blind test to provide a more realistic validation than the PDBbind v2007 benchmark. Its test set is composed of all the complexes in the PDBbind v2013 refined set that were not in the v2012 refined set, i.e. those 382 complexes that were newly added in the v2013 release. Its training set is simply the v2012 refined set, which contains 2897 complexes. By construction, this benchmark can be regarded as a blind test in that only data available until a certain year is used to build the scoring function that will be used to predict the binding affinity of future complexes as if these had not yet been measured. Consequently, the test set and training set do not overlap. Again, this benchmark has the advantage of permitting a direct comparison against the same four models that were trained and tested on crystal poses [6] of this benchmark.

In addition to the above training set, three more training sets were added in order to study how the performance of the four models would vary given different number of training complexes. The refined sets of PDBbind v2002 (N=792), v2007 (N=1300), v2010 (N=2057) and v2012 (N=2897) were chosen so that there is approximately the same number of complexes between consecutive releases. Complexes containing metal ions not supported by Vina were discarded. More details about this benchmark can be found in [6].

### Performance measures

As usual [15], predictive performance was quantified by the Root Mean Square Error (RMSE), Standard Deviation (SD), Pearson correlation (Rp) and Spearman rank-correlation (Rs) between predicted and measured binding affinities. Their mathematical expressions are shown in Eqs. 6, 7, 8, and 9. Given a scoring function *f* and the measured binding affinity *y*^(*n*)^ and the features $\overrightarrow {x}^{(n)}$ characterising the *n*th complex out of *N* complexes in the test set, $p^{(n)}=f(\overrightarrow {x}^{(n)})$ is the predicted binding affinity, $\{\hat {p}^{(n)}\}$ are the fitted values from the linear model between {*y*^(*n*)^} and {*p*^(*n*)^} on the test set, whereas $\{y_{r}^{(n)}\}$ and $\{p_{r}^{(n)}\}$ are the rankings of {*y*^(*n*)^} and {*p*^(*n*)^}, respectively. Note that SD was calculated in a linear correlation, but RMSE was not. Lower values in RMSE and SD and higher values in Rp and Rs indicate a better predictive performance.

6 $${} RMSE = \sqrt{\frac{1}{N}\sum\limits_{n=1}^{N}\left(p^{(n)}-y^{(n)}\right)^{2}}  $$

7 $${} SD = \sqrt{\frac{1}{N-2}\sum\limits_{n=1}^{N}\left(\hat{p}^{(n)}-y^{(n)}\right)^{2}}  $$

8 $${} {{\begin{aligned} R_{p} = \frac{N\sum_{n=1}^{N}p^{(n)}y^{(n)}-\sum_{n=1}^{N}p^{(n)}\sum_{n=1}^{N}y^{(n)}}{\sqrt{\!\left(\!N\sum_{n=1}^{N}(p^{(n)})^{2}-\left(\!\sum_{n=1}^{N}p^{(n)}\right)^{2}\!\right)\!\!\left(\!N\!\sum_{n=1}^{N}(y^{(n)})^{2}-\left(\!\sum_{n=1}^{N}y^{(n)}\right)^{2}\!\right)}} \end{aligned}}}  $$

9 $$ {{\begin{aligned} R_{s} = \frac{N\sum_{n=1}^{N}p_{r}^{(n)}y_{r}^{(n)}-\sum_{n=1}^{N}p_{r}^{(n)}\sum_{n=1}^{N}y_{r}^{(n)}}{\sqrt{\left(\!N\sum_{n=1}^{N}(p_{r}^{(n)})^{2}-\left(\!\sum_{n=1}^{N}p_{r}^{(n)}\!\right)^{2}\!\right)\!\!\left(\!N\sum_{n=1}^{N}(y_{r}^{(n)})^{2}-\left(\!\sum_{n=1}^{N}y_{r}^{(n)}\!\right)^{2}\!\right)}} \end{aligned}}}  $$

The Root Mean Square Deviation (RMSD) measures how geometrically different the redocked pose is from the corresponding co-crystallized pose of the same ligand molecule, i.e. the pose generation error. Suppose *N*_*a*_ is the number of heavy atoms, $\left (x_{c}^{(n)}, y_{c}^{(n)}, z_{c}^{(n)}\right)$ and $\left (x_{d}^{(n)}, y_{d}^{(n)}, z_{d}^{(n)}\right)$ are the 3D coordinate of the *n*th heavy atom of the crystal and docked poses, respectively, the pose generation error is calculated as:

10 $${} {{\begin{aligned} RMSD =\! \sqrt{\!\!\frac{1}{N_{a}}\sum_{n=1}^{N_{a}}\!\left[\!\left(\!x_{c}^{(n)}-x_{d}^{(n)}\!\right)^{2}\,+\,\left(\!y_{c}^{(n)}\,-\,y_{d}^{(n)}\!\right)^{2}\,+\,\left(\!z_{c}^{(n)}-z_{d}^{(n)}\!\right)^{2}\right]} \end{aligned}}}  $$

### Experimental setup

To generate docked poses, each ligand in the two benchmarks was docked into the binding site of its target protein using Vina with its default settings. This process is known as redocking. The search space was defined by finding the smallest cubic box that covers the entire ligand and then by extending the box by 10Å in X, Y, Z dimensions. Water molecules were removed, while metal ions recognized by Vina were retained as part of the protein. This preprocessing procedure is commonly adopted in the development of both classical scoring functions [1] and machine-learning scoring functions [16].

Redocking a ligand into its cognate protein resulted in up to nine docked poses. Thus, the question arises of which pose best represents its molecule for calculating the values of the features. Here we evaluate different schemes referring to the specific pose from which the features are extracted. In scheme 1, the chosen pose is the crystal pose. In scheme 2, the chosen pose is the docked pose with the best Vina score, i.e. the one with the lowest Vina score in terms of estimated free energy of binding in kcal/mol units. We trained the four models on both crystal and docked poses (in both schemes), and tested them also on both crystal and docked poses (in both schemes).

To make our experiments comprehensive, we also evaluated additional schemes. In scheme 3, the chosen pose is the docked pose with the lowest RMSD. In scheme 4, the chosen pose is the docked pose with a Vina score closest to the measured binding affinity. In scheme 5, the chosen poses are all the 9 docked poses, which hence results in a 9 times larger feature set (the number of features is 91 for models 2 and 3, and 415 for model 4). For ligands with less than 9 docked poses returned, the features extracted from the pose with the lowest Vina score are repeated as many times as poses are missing. In scheme 6, the chosen poses are the 2 docked poses with the lowest and the second lowest Vina score, which hence results in a double-sized feature set (the number of features is 21 for models 2 and 3, and 93 for model 4). For ligands with less than 2 docked poses outputted, the features extracted from the pose with the lowest Vina score are repeated. The rationale of introducing these schemes is that, schemes 1 to 4 help to determine which particular pose would be useful for improving predictive accuracy, while schemes 5 and 6 help to examine the effect of pose ensemble instead of a single pose.

Hereafter whenever we mention the docked pose, we implicitly refer to the one with the best Vina score (scheme 2), if not specified otherwise.

## Results

### Pose generation error slightly worsens binding affinity prediction

This question was analysed by using schemes 1 and 2. After redocking by Vina, we used RMSD to quantify the pose generation error, i.e. how different the 3D geometry of the redocked pose is from the corresponding crystal pose of the same ligand molecule. A RMSD value of 2Å was used as a commonly accepted threshold for a correctly reproduced crystal pose. 101 out of the 195 ligands (52 %) in the PDBbind v2007 benchmark and 219 out of the 382 ligands (57 %) in the PDBbind v2013 blind benchmark had their best-scoring docked pose with RMSD < 2Å. When all the docked poses of the molecule were considered, the redocking success rate of the two benchmarks increased to 76 % (149 out of 195) and 81 % (311 out of 382), respectively. These results are consistent with the previous results obtained in [2], where Vina managed to predict a pose sufficiently close to that of the co-crystallized ligand as the best-scoring pose in over half of the cases.

**Fig. 2:**
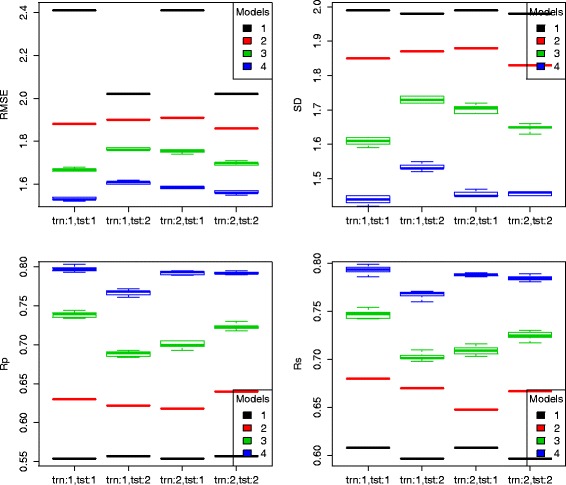
Box plots of performance of the four models trained on crystal and docked poses and tested also on crystal and docked poses of the PDBbind v2007 benchmark. Model 1 is AutoDock Vina, model 2 is MLR::Vina, model 3 is RF::Vina, and model 4 is RF::VinaElem. In the x axis, trn:1 means the four models were trained in scheme 1, i.e. on crystal poses, and trn:2 means the four models were trained in scheme 2, i.e. on docked poses. Likewise, tst-1 and tst-2 mean the four models were tested on crystal and docked poses, respectively. Model 1 was executed out-of-the-box, so its results for training scheme 1 were repeated for training scheme 2

**Fig. 3:**
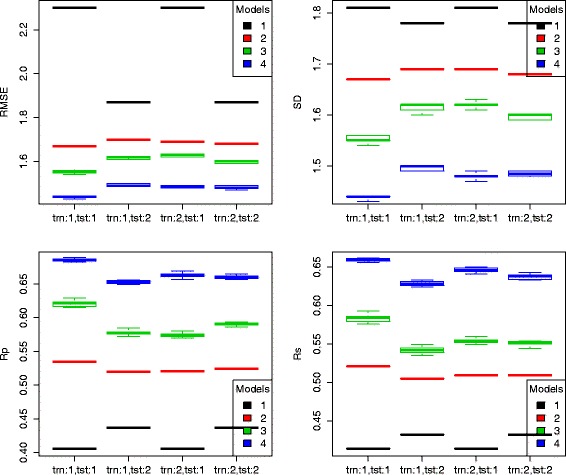
Box plots of performance of the four models trained on crystal and docked poses and tested also on crystal and docked poses of the PDBbind v2013 blind benchmark. The same notations are applied here as in Fig. 2

Tables 1 and 2 show the predictive performance of the four models trained on crystal and docked poses and tested also on crystal and docked poses on the PDBbind v2007 benchmark and the PDBbind v2013 blind benchmark, respectively. Figures 2 and 3 visualize the same results using boxplots, as RF models are stochastic. Note that Vina (model 1) was trained on crystal poses and used out-of-the-box without re-training, so its results for training scheme 2 are simply a duplicate of its results for training scheme 1.

**Table 1:** Performance of the four models trained on crystal and docked poses and tested also on crystal and docked poses (schemes 1 and 2) on the PDBbind v2007 benchmark. Comparing the same models from the two first blocks (crystal:crystal and crystal:docked) shows that the pose generation error also introduces a small degradation in the test set performance. Making the same comparisons between the second and fourth blocks shows that a substantial part of this error has been corrected

Model	Training	Test	RMSE	SD	Rp	Rs
1 (Vina)	Crystal	Crystal	2.41	1.99	0.554	0.608
2 (MLR::Vina)	Crystal	Crystal	1.88	1.85	0.630	0.680
3 (RF::Vina)	Crystal	Crystal	1.66	1.59	0.744	0.752
4 (RF::VinaElem)	Crystal	Crystal	1.52	1.42	0.803	0.799
1 (Vina)	Crystal	Docked	2.02	1.98	0.557	0.597
2 (MLR::Vina)	Crystal	Docked	1.90	1.87	0.622	0.670
3 (RF::Vina)	Crystal	Docked	1.76	1.72	0.693	0.710
4 (RF::VinaElem)	Crystal	Docked	1.60	1.52	0.772	0.771
2 (MLR::Vina)	Docked	Crystal	1.91	1.88	0.618	0.648
3 (RF::Vina)	Docked	Crystal	1.74	1.69	0.705	0.716
4 (RF::VinaElem)	Docked	Crystal	1.58	1.45	0.794	0.790
2 (MLR::Vina)	Docked	Docked	1.86	1.83	0.640	0.667
3 (RF::Vina)	Docked	Docked	1.69	1.63	0.730	0.730
4 (RF::VinaElem)	Docked	Docked	1.55	1.45	0.795	0.789

**Table 2:** Performance of the four models trained on crystal and docked poses and tested also on crystal and docked poses (schemes 1 and 2) on the PDBbind v2013 blind benchmark

Model	Training	Test	RMSE	SD	Rp	Rs
1 (Vina)	Crystal	Crystal	2.30	1.81	0.406	0.414
2 (MLR::Vina)	Crystal	Crystal	1.67	1.67	0.535	0.521
3 (RF::Vina)	Crystal	Crystal	1.54	1.54	0.629	0.593
4 (RF::VinaElem)	Crystal	Crystal	1.43	1.43	0.689	0.662
1 (Vina)	Crystal	Docked	1.87	1.78	0.437	0.432
2 (MLR::Vina)	Crystal	Docked	1.70	1.69	0.520	0.505
3 (RF::Vina)	Crystal	Docked	1.61	1.60	0.585	0.549
4 (RF::VinaElem)	Crystal	Docked	1.49	1.49	0.656	0.633
2 (MLR::Vina)	Docked	Crystal	1.69	1.69	0.521	0.509
3 (RF::Vina)	Docked	Crystal	1.62	1.61	0.580	0.560
4 (RF::VinaElem)	Docked	Crystal	1.48	1.47	0.669	0.650
2 (MLR::Vina)	Docked	Docked	1.68	1.68	0.524	0.509
3 (RF::Vina)	Docked	Docked	1.59	1.59	0.594	0.553
4 (RF::VinaElem)	Docked	Docked	1.47	1.48	0.665	0.643

From these results, several interesting observations can be made. First, for model 1, its performance tested on docked poses was always better than its performance tested on crystal poses (except for just a small degradation in the Rs performance on the PDBbind v2007 benchmark). Particularly, the RMSE error was greatly dropped from 2.41 to 2.02 on the PDBbind v2007 benchmark and from 2.30 to 1.87 on the PDBbind v2013 blind benchmark. The result that Vina made better prediction of binding affinity from docked poses than from crystal poses is possibly due to the fact that docked poses are by construction optima of the objective function spanned by the Vina score, which may favor prediction of docked poses over unoptimized crystal poses.

Second, for models 2, 3 and 4 trained on crystal poses, their performance tested on docked poses was always worse than their performance tested on crystal poses (e.g. by comparing second and first columns in the Rs plot of Fig. 3). This is well anticipated because of the presence of pose generation error. For instance, on the PDBbind v2013 blind benchmark, model 4 trained on crystal poses obtained Rs=0.662 when tested on crystal poses (Additional file 1). Its performance degraded when tested on docked poses of the same molecules with Rs=0.633 (Additional file 2). The impact of pose generation error on binding affinity prediction is thus quantified by *Δ*Rs=-0.029.

Third, for models 2, 3 and 4 tested on docked poses, their performance was better when they were trained on docked poses than their counterparts trained on crystal poses (e.g. by comparing fourth and second columns in the Rs plot of Fig. 3). In other words, a substantial part of the pose generation was corrected. For instance, on the PDBbind v2013 blind benchmark, model 4 trained on docked poses obtained Rs=0.643 when tested on docked poses (Additional file 3). Hence the impact of pose generation error on binding affinity prediction is reduced in a 33 % (from *Δ*Rs=-0.029 to *Δ*Rs=-0.019). This means that a way to improve performance on docked poses is to train the model on docked poses instead of on crystal poses. Indeed, test set performance after this error-correcting procedure is much closer to that of predicting the binding affinity in the absence of pose generation error, i.e. on crystal structures. In practice, different scoring functions can be built depending on whether one wants to score crystal poses or docked poses.

Fourth, for models 2, 3 and 4 tested on crystal poses, the models trained on docked poses (Additional file 4) did not outperform their counterparts trained on crystal poses. This is also well anticipated due to the impact of pose generation error, and suggests that it is not feasible to improve the predictive performance on crystal poses by using docked poses for training. To sum up, if the desired application is to score a crystal pose, it would be better to train the scoring function on crystal poses; and if the desired application is to score a docked pose, it would be better to train the scoring function on docked poses.

Lastly, regardless of the training or test schemes, model 4 consistently outperformed model 3, which in turn outperformed model 2, which in turn outperformed model 1. It is remarkable that the best scoring function, model 4 (RF::VinaElem), when trained on docked poses, achieved the highest performance in the literature on the PDBbind v2007 benchmark in the more common application of re-scoring docked poses, as it is required when carrying out docking-based prospective virtual screening [2]. Here we denote this version of RF::VinaElem as RF-Score-v4 specifically for the purpose of binding affinity prediction given a docked pose from Vina. Importantly, since Vina and RF::Vina used the same features and were trained on the same data, RF::Vina performed much better in predicting binding affinity than the widely-used Vina software while having the same applicability domain.

### Training with more complexes on docked poses still improves predictive performance

**Fig. 4:**
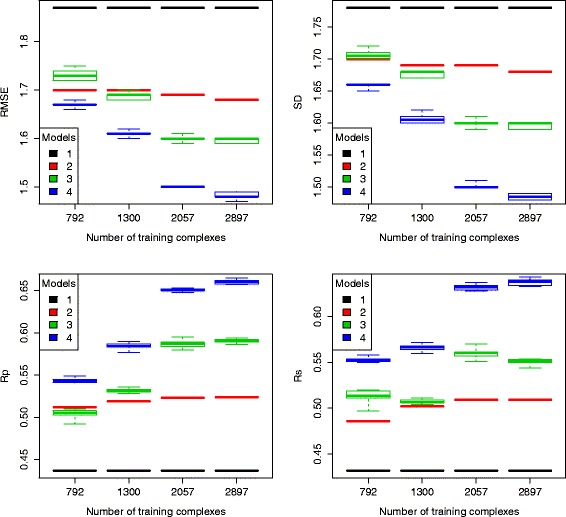
Box plots of performance of the four models trained on docked poses and tested also on docked poses of the PDBbind v2013 blind benchmark, with four incrementally-sized training sets. Model 1 is AutoDock Vina, model 2 is MLR::Vina, model 3 is RF::Vina, and model 4 is RF::VinaElem

In a previous study [6], with the help of the PDBbind v2013 blind benchmark, we showed that training RF models with larger datasets greatly improved their predictive performance on scoring crystal poses, while the performance of MLR::Vina nearly stayed flat. Here we observe similar results when the models were trained on docked poses and tested also on docked poses. As shown in Fig. 4, when more complexes were used for model training, RF::VinaElem consistently increased its predictive accuracy in terms of RMSE, SD, Rp and Rs. In contrast, for MLR::Vina, its accuracy improvement obtained from larger training sets was just marginal, if not negligible. The performance gap between MLR::Vina and RF::VinaElem is not only substantial, but grows as more data is available for training, thus increasing the importance of employing RF in scoring function development. More importantly, the availability of crystal poses is limited by the number of experimentally resolved structures, whereas docked poses can be generated by docking tools if their binding site is known. This means that, by using docked poses for training, the training data size can be remarkably larger than limiting the training data to crystal poses only, and therefore even higher performance could in principle be achieved by incorporating more training complexes produced by docking.

### Correlation between pose generation error and binding affinity prediction error is low

We analyse how different RMSD values affect binding affinity prediction by comparing the RMSD of the docked pose with the individual absolute error in its binding affinity prediction by the four models (note that the square root of the summation of the square of these errors is the RMSE measure). It is widely believed that the higher the pose generation error, the larger the error on predicting the binding affinity of that pose will be. Nevertheless, this is actually not the case.

**Fig. 5:**
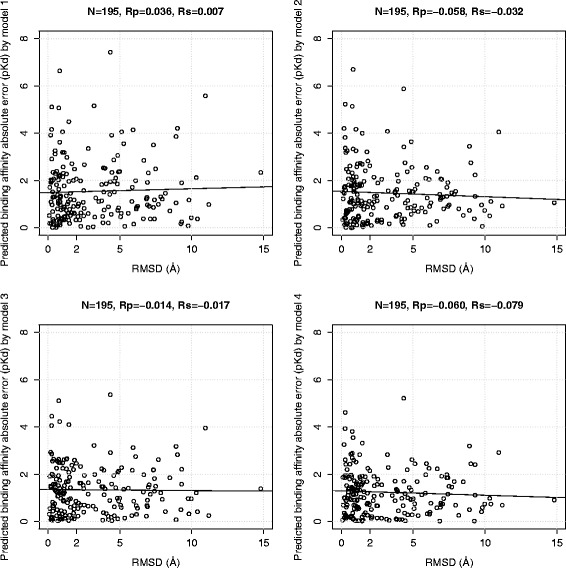
Correlation plots of predicted binding affinity absolute errors achieved by the four models trained on docked poses and tested on docked poses of the PDBbind v2007 benchmark against the RMSD values from redocking the 195 test set complexes by Vina. Model 1 is AutoDock Vina, model 2 is MLR::Vina, model 3 is RF::Vina, and model 4 is RF::VinaElem

**Fig. 6:**
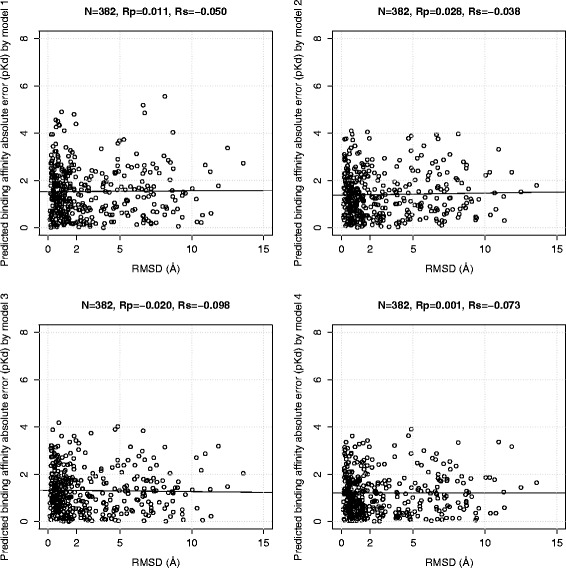
Correlation plots of predicted binding affinity absolute errors achieved by the four models trained on docked poses and tested on docked poses of the PDBbind v2013 blind benchmark against the RMSD values from redocking the 382 test set complexes by Vina. Model 1 is AutoDock Vina, model 2 is MLR::Vina, model 3 is RF::Vina, and model 4 is RF::VinaElem

Figures 5 and 6 plot this information for each of the four scoring functions trained and tested on docked poses of the two benchmarks, respectively. Strikingly, the four scoring functions are particularly robust to pose generation error, with reasonably accurate prediction still being obtained in poses with RMSD of almost 15Å. The Rp and Rs values stated at the top of these plots quantify how little the pose generation error generally correlates with the binding affinity prediction error, regardless of whether a classical or machine-learning scoring function is being considered. This is likely to be connected to uncertainty associated to relating a static crystal structure of the complex with its measured binding affinity which is the outcome of the dynamic process of binding, as discussed in [5]. To the best of our knowledge, these behaviour has not been communicated yet for classical scoring functions, which is highly surprising given the intense research that has been carried out over the years in this area. On the other hand, it is noteworthy that, while the binding affinities of some complexes are very well predicted (*pK*_*d*_ error close to 0), some others have errors of more than 7 *pK*_*d*_ units (see the topleft plots for Vina).

### Using multiple docked poses for training does not improve predictive performance

In addition to using crystal and docked poses (schemes 1 and 2) for training and testing, we further evaluated several strategies (schemes 3, 4, 5 and 6), aiming to see if using another docked pose of a molecule, or even multiple docked poses, could possibly increase the predictive performance of the resultant models. Remember that scheme 3 uses the docked pose with the lowest RMSD, scheme 4 uses the docked pose with a Vina score closest to the measured binding affinity, scheme 5 uses all the 9 docked poses, and scheme 6 uses the two top-scoring docked poses. In practice, schemes 3 and 4 cannot be used for testing purpose because neither the RMSD nor the measured binding affinity of the test set complexes are known. Hence, models trained in schemes 3 and 4 had to be tested in schemes 1 and 2 instead. On the other hand, models trained in schemes 5 and 6 can only be tested in schemes 5 and 6, respectively, because the same set of features must be used in both training and testing.

The results of schemes 3 to 6 on the two benchmarks are shown in Tables 3 and 4. Interestingly, when tested on crystal poses (in scheme 1), none of the models trained in schemes 3 to 6 outperformed their counterparts trained in scheme 1. Similarly, when tested on docked poses (in scheme 2), none of the models trained in schemes 3 to 6 outperformed their counterparts trained in scheme 2, either. The interpretation of such results is two-fold. First, training with the docked pose with the lowest RMSD (scheme 3) or the docked pose with a Vina score closest the measured binding affinity (scheme 4) did not help to improve predictive performance on the test set. Second, training with multiple docked poses of a molecule, instead of a specific single docked pose, did not help to improve predictive performance either. Taken together, these results suggest that a novel way to improve predictive performance on docked poses is to train the scoring functions on docked poses, i.e. those with the best Vina score.

**Table 3:** Performance of models 2, 3, 4 trained in schemes 3, 4, 5, 6 and tested in schemes 1, 2, 5, 6 on the PDBbind v2007 benchmark

Model	Training	Test	RMSE	SD	Rp	Rs
2 (MLR::Vina)	3	1	1.89	1.85	0.629	0.675
3 (RF::Vina)	3	1	1.76	1.73	0.691	0.694
4 (RF::VinaElem)	3	1	1.58	1.45	0.795	0.792
2 (MLR::Vina)	3	2	1.88	1.85	0.630	0.661
3 (RF::Vina)	3	2	1.72	1.68	0.711	0.714
4 (RF::VinaElem)	3	2	1.57	1.45	0.793	0.780
2 (MLR::Vina)	4	1	1.93	1.93	0.589	0.648
3 (RF::Vina)	4	1	1.81	1.80	0.656	0.669
4 (RF::VinaElem)	4	1	1.63	1.53	0.769	0.769
2 (MLR::Vina)	4	2	1.94	1.93	0.589	0.636
3 (RF::Vina)	4	2	1.79	1.75	0.682	0.686
4 (RF::VinaElem)	4	2	1.63	1.53	0.769	0.762
2 (MLR::Vina)	5	5	1.90	1.89	0.609	0.641
3 (RF::Vina)	5	5	1.74	1.70	0.700	0.699
4 (RF::VinaElem)	5	5	1.65	1.55	0.760	0.754
2 (MLR::Vina)	6	6	1.86	1.83	0.640	0.670
3 (RF::Vina)	6	6	1.73	1.69	0.707	0.707
4 (RF::VinaElem)	6	6	1.60	1.49	0.780	0.769

**Table 4:** Performance of models 2, 3, 4 trained in schemes 3, 4, 5, 6 and tested in schemes 1, 2, 5, 6 on the PDBbind v2013 blind benchmark

Model	Training	Test	RMSE	SD	Rp	Rs
2 (MLR::Vina)	3	1	1.70	1.69	0.521	0.511
3 (RF::Vina)	3	1	1.60	1.58	0.602	0.575
4 (RF::VinaElem)	3	1	1.48	1.48	0.666	0.643
2 (MLR::Vina)	3	2	1.69	1.69	0.523	0.509
3 (RF::Vina)	3	2	1.59	1.58	0.601	0.562
4 (RF::VinaElem)	3	2	1.49	1.49	0.655	0.635
2 (MLR::Vina)	4	1	1.88	1.80	0.413	0.415
3 (RF::Vina)	4	1	1.72	1.71	0.499	0.477
4 (RF::VinaElem)	4	1	1.57	1.57	0.610	0.589
2 (MLR::Vina)	4	2	1.77	1.75	0.468	0.447
3 (RF::Vina)	4	2	1.70	1.66	0.544	0.508
4 (RF::VinaElem)	4	2	1.58	1.57	0.611	0.582
2 (MLR::Vina)	5	5	1.65	1.65	0.550	0.526
3 (RF::Vina)	5	5	1.58	1.58	0.603	0.578
4 (RF::VinaElem)	5	5	1.49	1.50	0.653	0.633
2 (MLR::Vina)	6	6	1.68	1.68	0.526	0.514
3 (RF::Vina)	6	6	1.57	1.57	0.608	0.581
4 (RF::VinaElem)	6	6	1.47	1.48	0.665	0.643

## Discussions and conclusions

This is the first study that systematically investigates the impact of pose generation error on binding affinity prediction for both classical and machine-learning scoring functions. Our comprehensive results show that pose generation error only introduces a small degradation in the accuracy of scoring functions. To minimize this negative impact, we found that re-training the scoring functions on docked poses, instead of crystal poses, corrects a substantial part of this degradation. Machine-learning scoring functions are almost always trained on crystal poses and tested on crystal or docked poses without changing composition of training or test sets. Here we still tested the scoring functions on docked poses, but now trained them on docked poses, which has been shown to improve test set performance with respect to the scoring functions trained on crystal poses. In short, one straightforward approach to enhancing predictive accuracy on docked poses is to re-train the scoring function on docked poses.

We have also found that training RF::VinaElem on docked poses with more complexes substantially increased its predictive accuracy, whereas it was not the case for MLR::Vina. Their performance gap will become larger given that more and more structural and interaction data will be available for training in the future. Importantly, whereas the availability of crystal poses is limited by the number of experimentally resolved structures, docked poses of the many known ligands of these targets can be generated by docking tools if their binding site is known. This means that, by using docked poses for training, the training data size can be remarkably larger than limiting the training data to crystal poses only, and therefore even higher performance could in principle be achieved by incorporating more training complexes produced by docking.

Furthermore, we investigated the dependency of RMSD of test set complexes with binding affinity prediction. In contrast to the commonly-held view that the higher the pose generation error, the larger the prediction error of the binding affinity of that pose, we actually observed that the correlation between pose generation error and binding affinity prediction error is low. This indicates that predicting the binding affinity of a docked pose having a large pose generation error is not necessarily more difficult than predicting the binding affinity of a docked pose having a small pose generation error.

Meanwhile, we studied the effect of using docked pose ensemble of a molecule, in addition to merely a single pose, for training scoring functions. This is worth doing because until now existing scoring functions all use just one pose per molecule for training. Although our presented schemes 3 to 6 did not succeed in increasing predictive performance, further analysis of the influence of the number of poses on the error in binding affinity prediction might lead to better performance.

Another contribution of this study is the release of free software implementing RF::VinaElem trained on docked poses, named RF-Score-v4, so that it can be directly used by the large number of Vina users (poses generated from other docking programs can also be re-scored by our software once these are converted to AutoDock’s pdbqt format). With this purpose, we have trained the best of RF-Score-v4 on the most comprehensive set of high-quality complexes (the 3441 complexes from the PDBbind v2014 refined set) and implemented it as easy-to-use software that directly re-scores Vina-generated poses. See the abstract for availability and the README file therein for operating instructions.

Last but not the least, although we only used RF in this study as a proof of concept, we believe our conclusions can be applicable to other machine-learning scoring functions, which could possibly achieve even better results on this problem.

## Additional files

Additional file 1 Correlation plots of measured and predicted binding affinities by the four models trained on crystal poses and tested on crystal poses of the PDBbind v2013 blind benchmark. (PDF 15 kb)

Additional file 2 Correlation plots of measured and predicted binding affinities by the four models trained on crystal poses and tested on docked poses of the PDBbind v2013 blind benchmark. (PDF 15 kb)

Additional file 3 Correlation plots of measured and predicted binding affinities by the four models trained on docked poses and tested on docked poses of the PDBbind v2013 blind benchmark. (PDf 15 kb)

Additional file 4 Correlation plots of measured and predicted binding affinities by the four models trained on docked poses and tested on crystal poses of the PDBbind v2013 blind benchmark. (PDF 15 kb)
