# Supplementary figures and images for: Correcting the impact of docking pose generation error on binding affinity prediction
Source: BMC Bioinformatics. 2016 Sep 22;17(Suppl 11):308. doi: 10.1186/s12859-016-1169-4 (PMC5046193; doi:10.1186/s12859-016-1169-4)

**N=382, RMSE=2.30, SD=1.81, Rp=0.406, Rs=0.414**

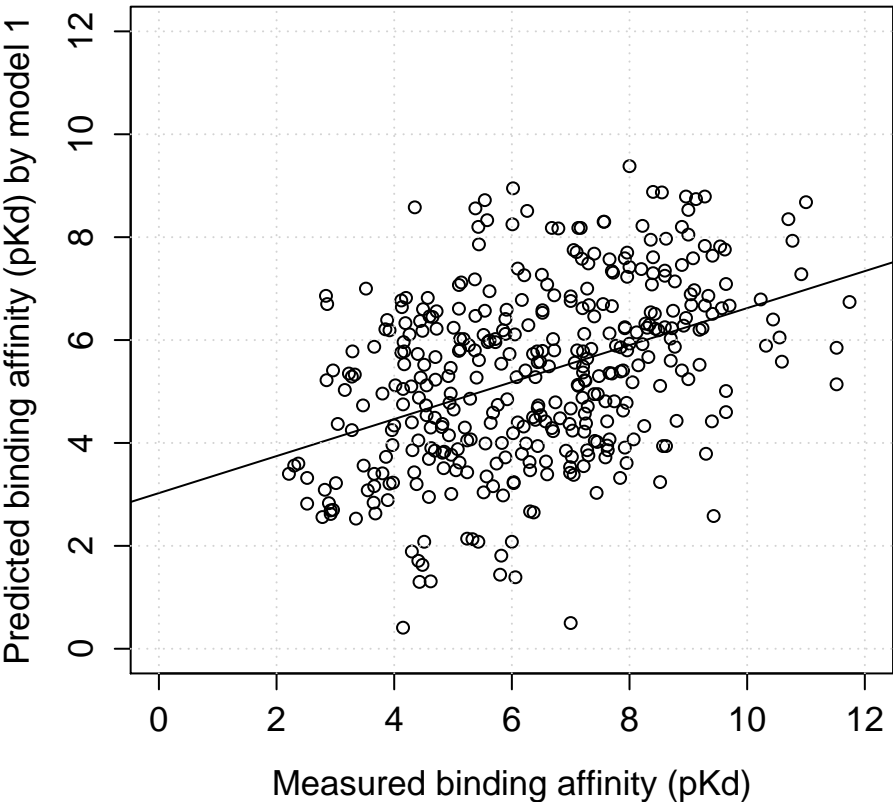

**N=382, RMSE=1.67, SD=1.67, Rp=0.535, Rs=0.521**

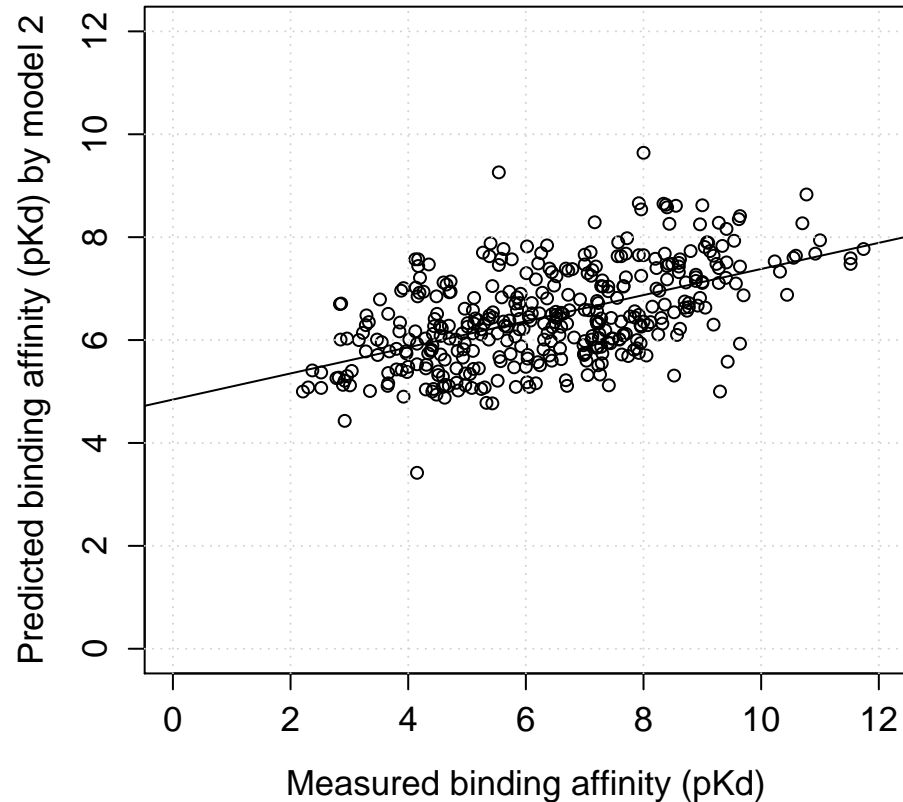

**N=382, RMSE=1.54, SD=1.54, Rp=0.629, Rs=0.593**

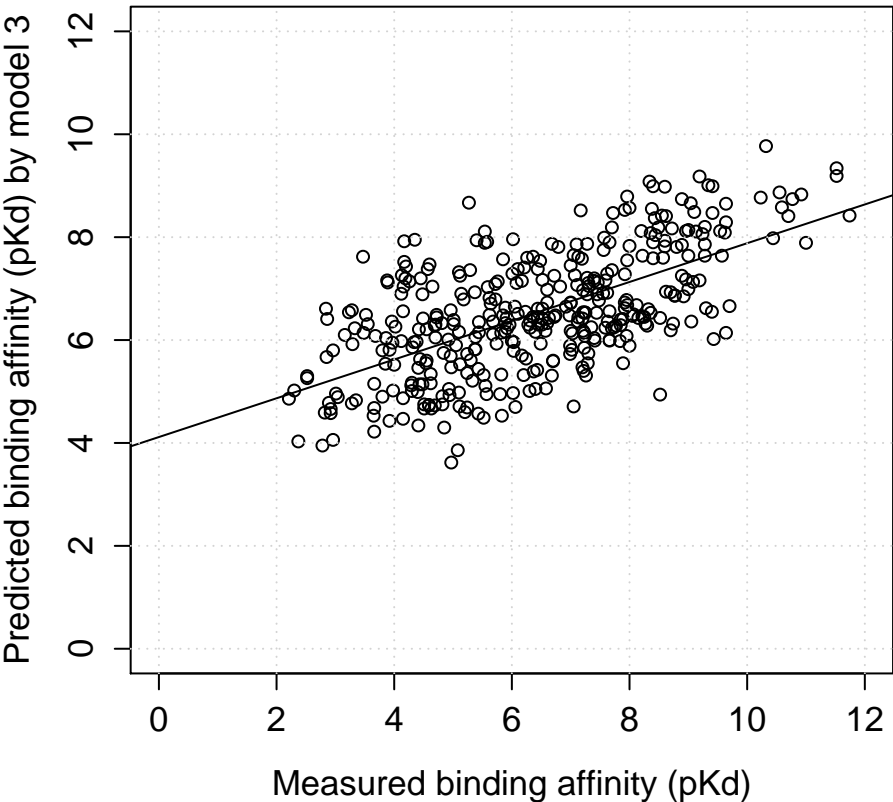

**N=382, RMSE=1.43, SD=1.43, Rp=0.689, Rs=0.662**

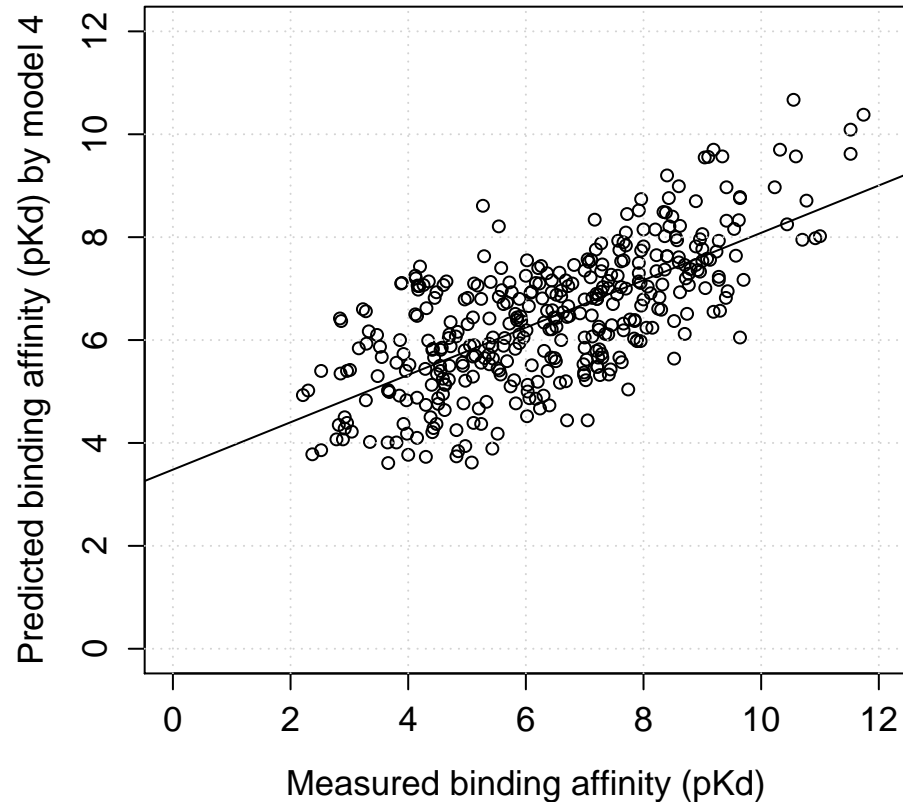

Supplement: Additional file 1 — Correlation plots of measured and predicted binding affinities by the four models trained on crystal poses and tested on crystal poses of the PDBbind v2013 blind benchmark. (PDF 15 kb) [file 12859_2016_1169_MOESM1_ESM.pdf]

**N=382, RMSE=1.87, SD=1.78, Rp=0.437, Rs=0.432**

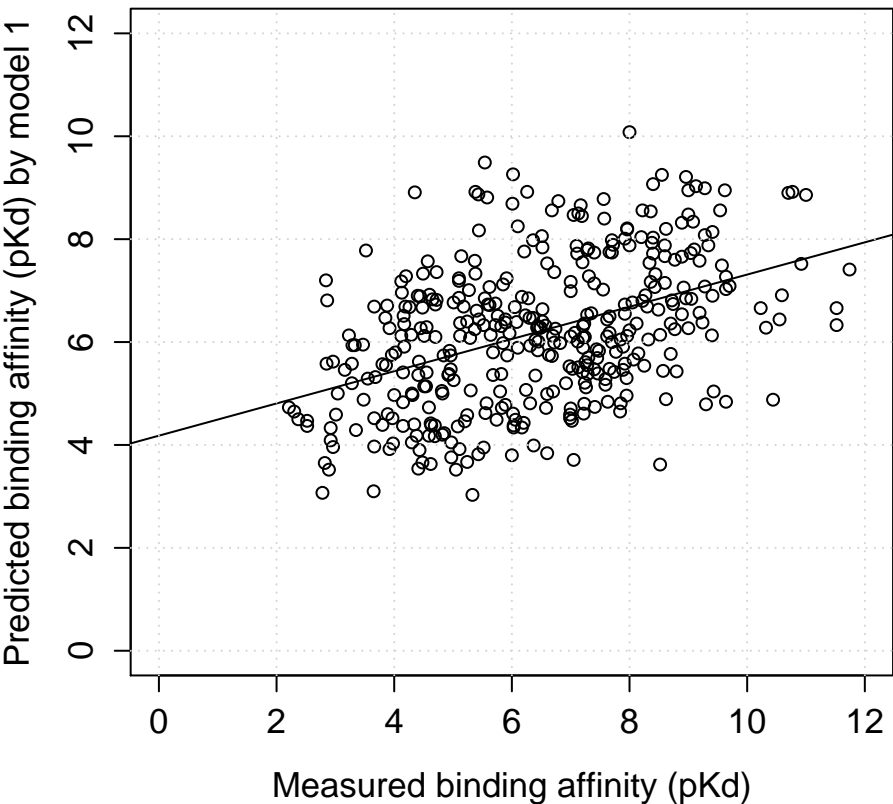

**N=382, RMSE=1.70, SD=1.69, Rp=0.520, Rs=0.505**

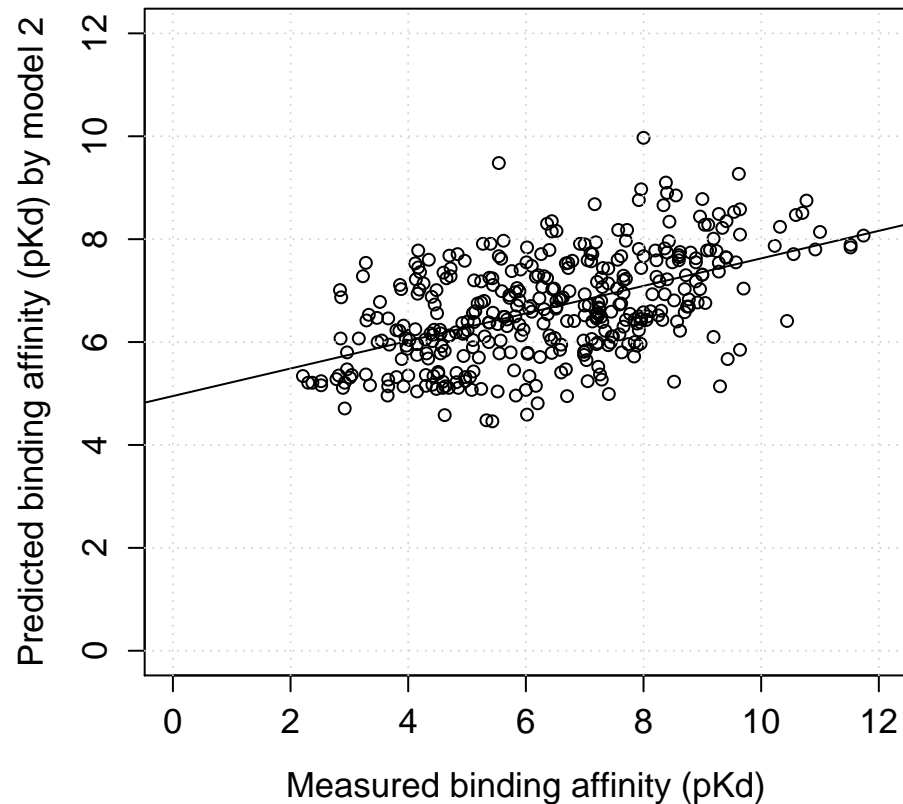

**N=382, RMSE=1.61, SD=1.60, Rp=0.585, Rs=0.549**

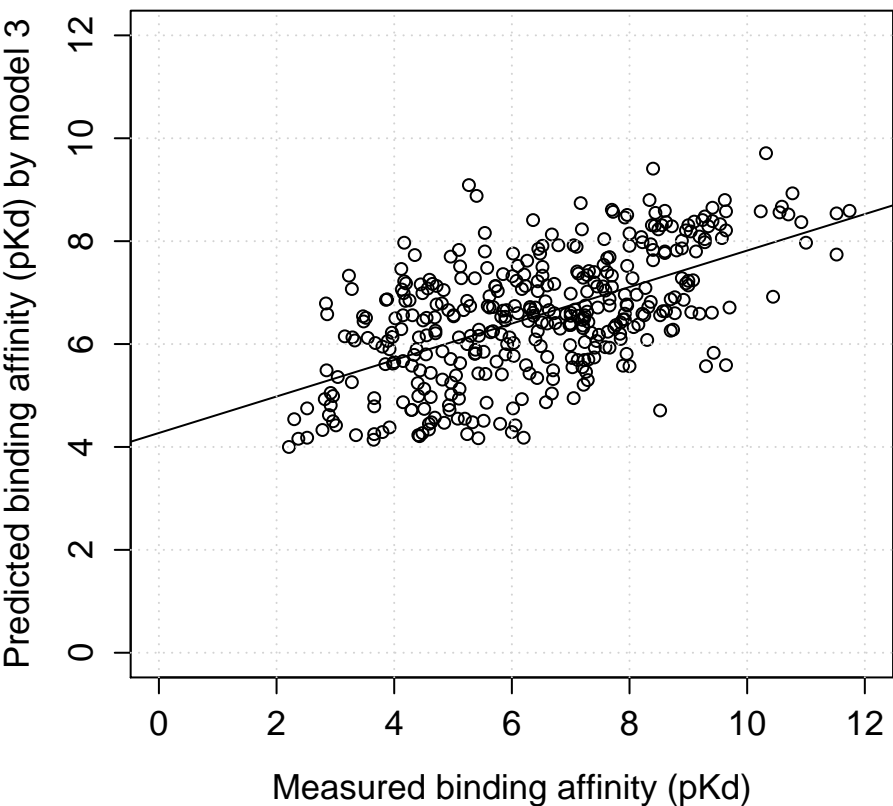

**N=382, RMSE=1.49, SD=1.49, Rp=0.656, Rs=0.633**

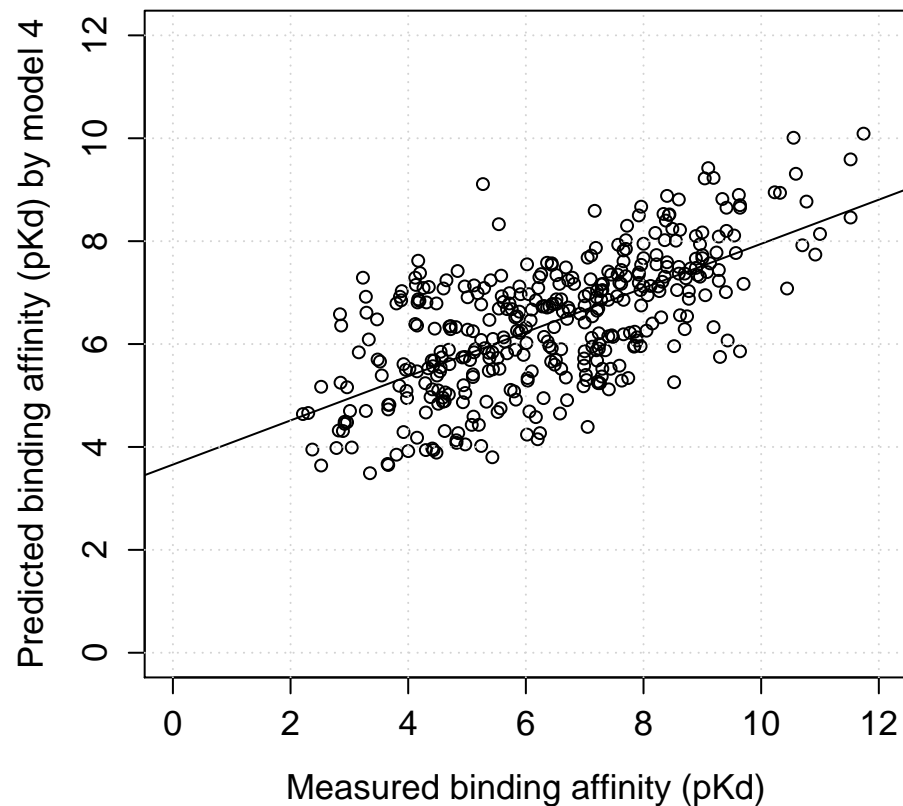

Supplement: Additional file 2 — Correlation plots of measured and predicted binding affinities by the four models trained on crystal poses and tested on docked poses of the PDBbind v2013 blind benchmark. (PDF 15 kb) [file 12859_2016_1169_MOESM2_ESM.pdf]

**N=382, RMSE=1.87, SD=1.78, Rp=0.437, Rs=0.432**

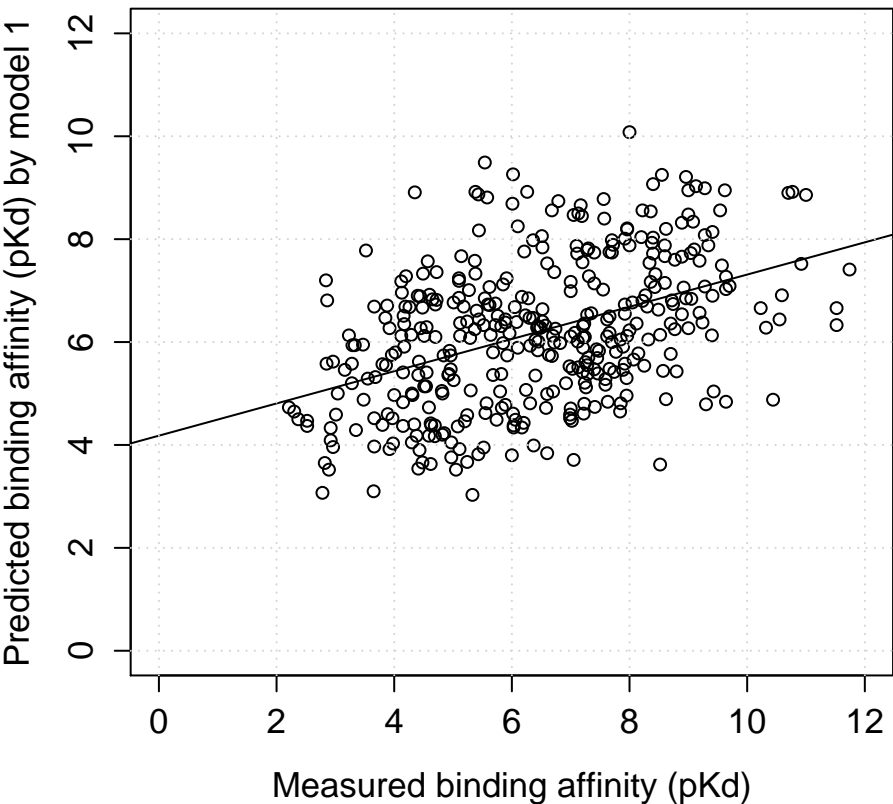

**N=382, RMSE=1.68, SD=1.68, Rp=0.524, Rs=0.509**

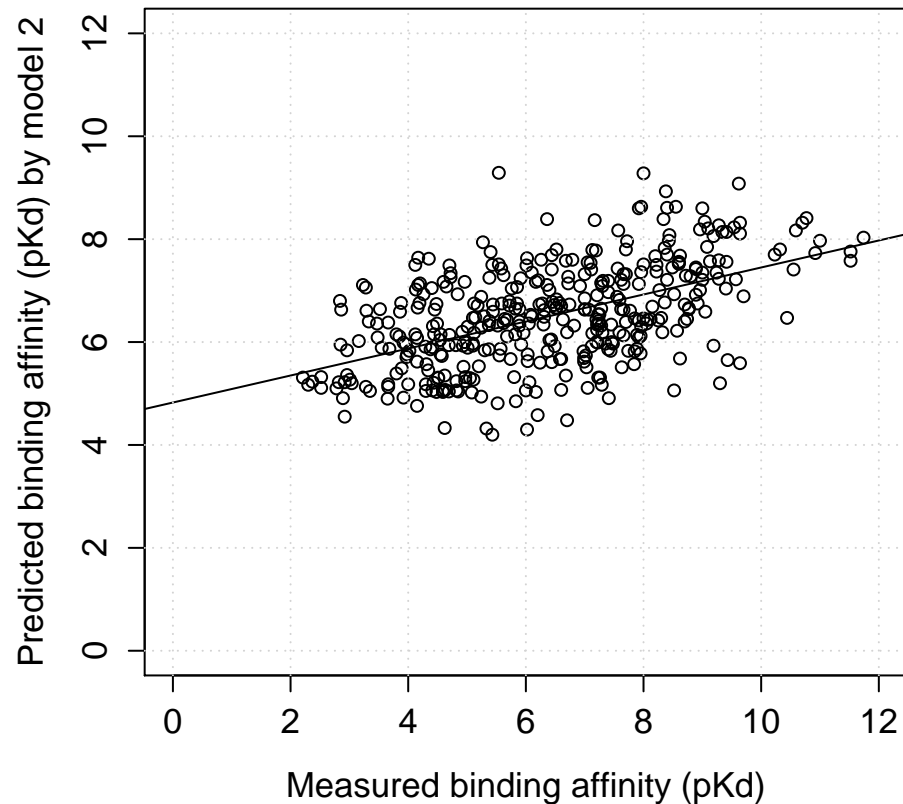

**N=382, RMSE=1.59, SD=1.59, Rp=0.594, Rs=0.553**

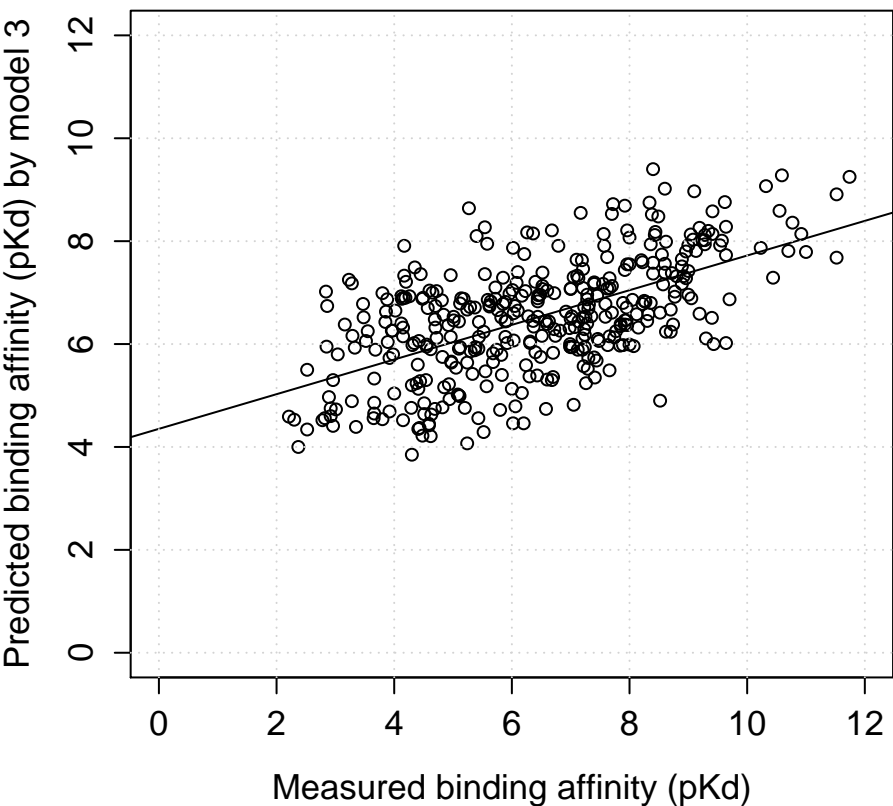

**N=382, RMSE=1.47, SD=1.48, Rp=0.665, Rs=0.643**

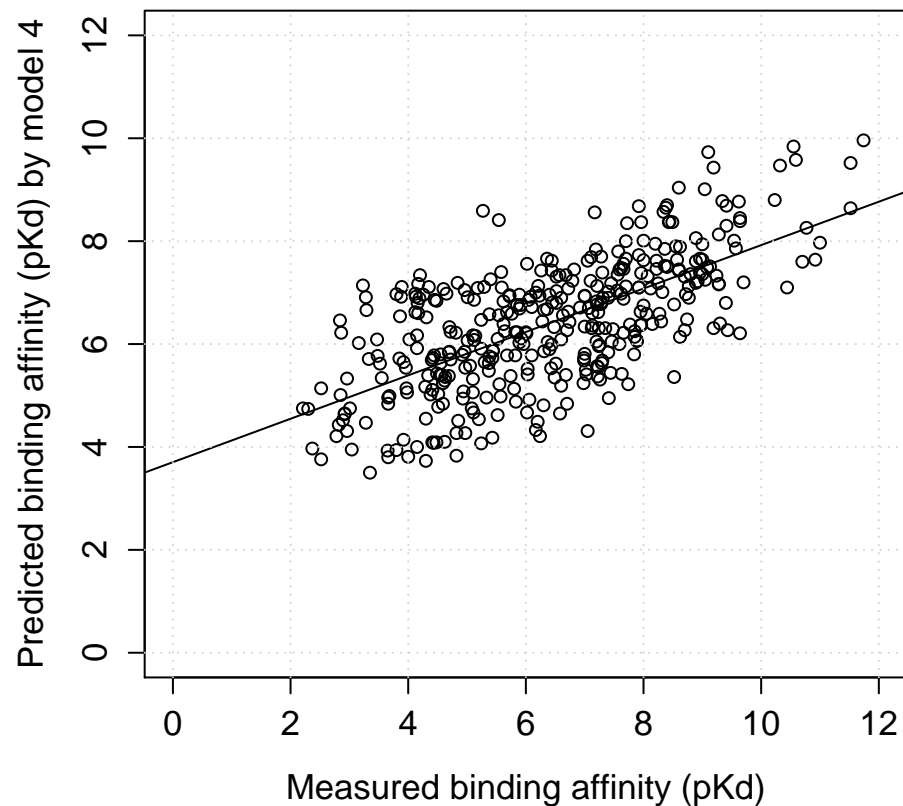

Supplement: Additional file 3 — Correlation plots of measured and predicted binding affinities by the four models trained on docked poses and tested on docked poses of the PDBbind v2013 blind benchmark. (PDf 15 kb) [file 12859_2016_1169_MOESM3_ESM.pdf]

**N=382, RMSE=2.30, SD=1.81, Rp=0.406, Rs=0.414**

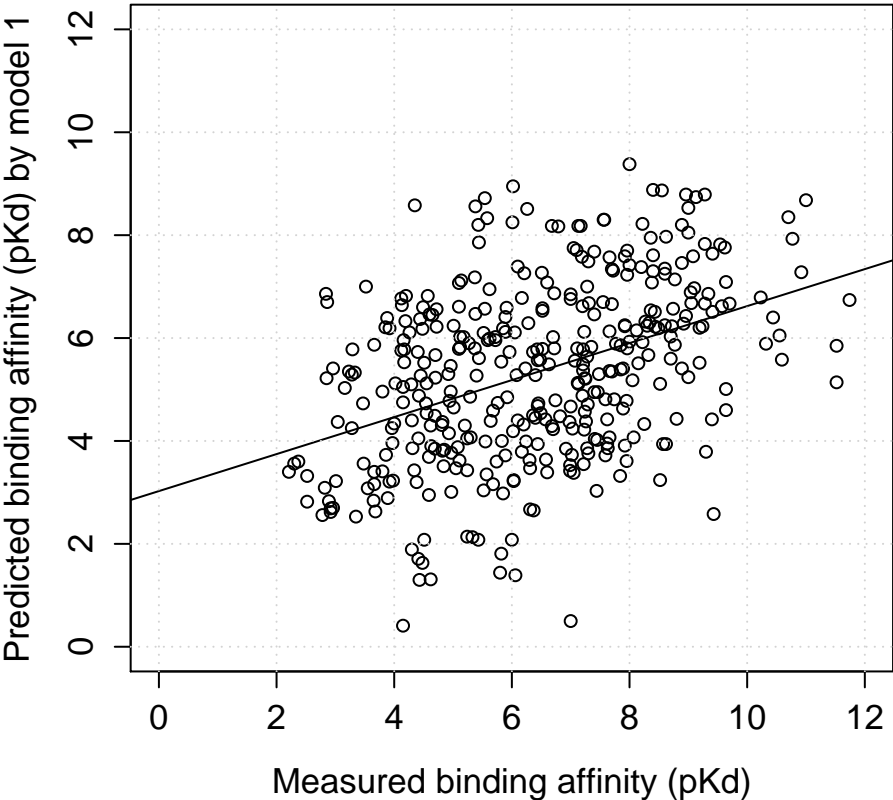

**N=382, RMSE=1.69, SD=1.69, Rp=0.521, Rs=0.509**

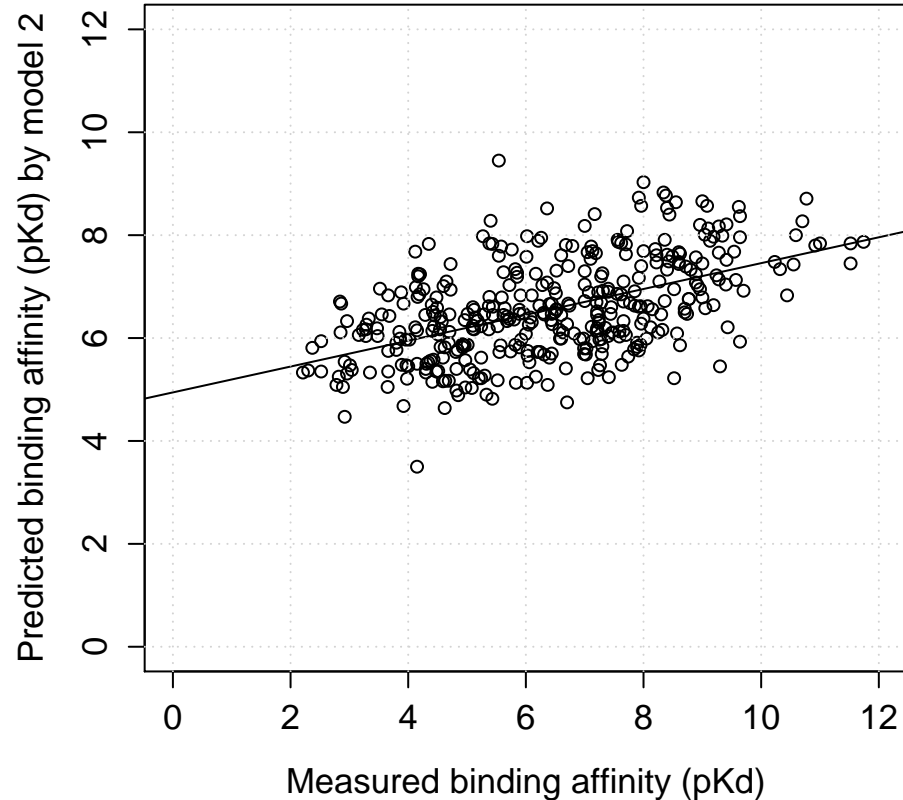

**N=382, RMSE=1.62, SD=1.61, Rp=0.580, Rs=0.560**

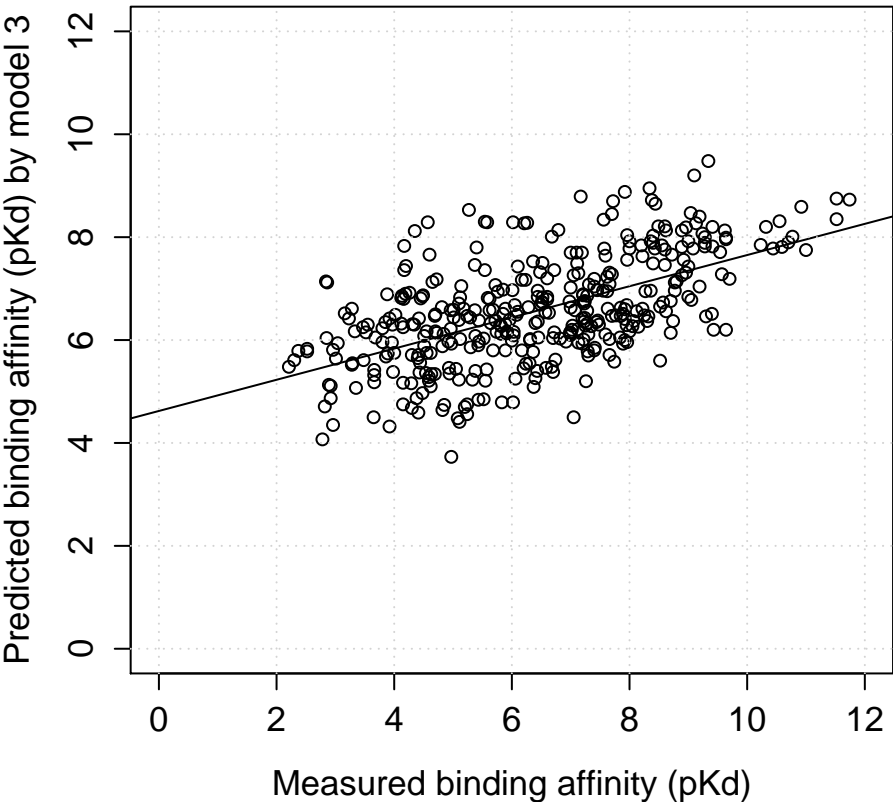

**N=382, RMSE=1.48, SD=1.47, Rp=0.669, Rs=0.650**

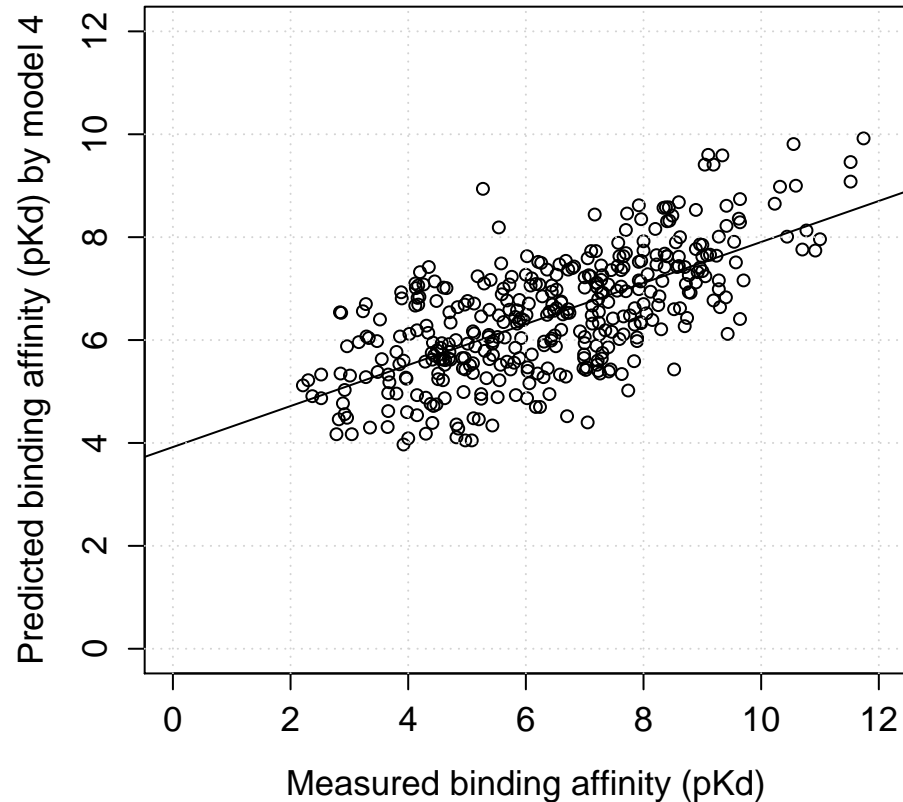

Supplement: Additional file 4 — Correlation plots of measured and predicted binding affinities by the four models trained on docked poses and tested on crystal poses of the PDBbind v2013 blind benchmark. (PDF 15 kb) [file 12859_2016_1169_MOESM4_ESM.pdf]
